# Recruitment of Polo Kinase to the Spindle Midzone during Cytokinesis Requires the Feo/Klp3A Complex

**DOI:** 10.1371/journal.pone.0000572

**Published:** 2007-06-27

**Authors:** Pier Paolo D'Avino, Vincent Archambault, Marcin R. Przewloka, Wei Zhang, Kathryn S. Lilley, Ernest Laue, David M. Glover

**Affiliations:** 1 Cancer Research UK Cell Cycle Genetics Research Group, Department of Genetics, University of Cambridge, Cambridge, United Kingdom; 2 Department of Biochemistry, University of Cambridge, Cambridge, United Kingdom; Institute for Research in Biomedicine (IRB), Barcelona, Spain

## Abstract

**Background:**

Polo-like kinases control multiple events during cell division, including mitotic entry, centrosome organization, spindle formation, chromosome segregation and cytokinesis. Their roles during cytokinesis, however, are not well understood because the requirement of these kinases during early stages of mitosis complicates the study of their functions after anaphase onset.

**Methodology/Principal Findings:**

We used time-lapse microscopy to analyze the dynamics of Polo::GFP in *Drosophila* tissue culture cells during mitosis. After anaphase onset, Polo::GFP concentrated at the spindle midzone, but also diffused along the entire length of the central spindle. Using RNA interference we demonstrate that the microtubule-associated proteins Feo and Klp3A are required for Polo recruitment to the spindle midzone, but not the kinesin Pavarotti as previously thought. Moreover, we show that Feo and Klp3A form a complex and that Polo co-localizes with both proteins during cytokinesis.

**Conclusion/Significance:**

Our results reveal that the Feo/Klp3A complex is necessary for Polo recruitment to the spindle midzone. A similar finding has also been recently reported in mammalian cells [1], suggesting that this basic mechanism has been conserved during evolution, albeit with some differences. Finally, since cleavage furrow formation and ingression are unaffected following *feo* RNAi, our data imply that Polo recruitment to the central spindle is not required for furrowing, but some other aspect of cytokinesis.

## Introduction

The formation and ingression of the cleavage furrow during cytokinesis depends on the co-ordinate actions of the microtubule and actomyosin cytoskeletons. Spindle microtubules dictate the position of the cleavage plane at the metaphase/anaphase transition and then reorganize into bundles of antiparallel and interdigitating microtubules known as the central spindle during cytokinesis [2], [3]. This microtubule structure is crucial for successful cytokinesis in many systems and several factors necessary for its proper assembly have been identified. These consist mostly of microtubule-associated proteins (MAPs), including kinesins, and signaling proteins such as protein kinases and phosphatases [3].

In mammals, the cyclin-dependent kinase 1 (CDK1) prevents central spindle assembly prior to anaphase onset by inhibiting the activity of at least two MAP proteins: MKLP1 and PRC1 [4], [5]. MKLP-1 is a highly conserved plus-end directed motor protein known as Pavarotti (Pav) in *Drosophila* and ZEN-4 in *C. elegans* [6], [7], [8], [9]. This kinesin is one of the two components of an evolutionary conserved complex, dubbed centralspindlin, which is essential for central spindle assembly in all metazoans [10], [11]. Phosphorylation of MKLP1 by CDK1 inhibits its ability to bind microtubules. Thus, only after inactivation of CDK1 by the anaphase-promoting complex (APC) can centralspindlin translocate to the plus ends of microtubules where it promotes central spindle assembly [5]. Similarly, CDK1 phosphorylation of PRC1 (Fascetto in *Drosophila* and SPD-1 in *C. elegans*) prevents its interaction with the kinesin KIF-4 and consequent localization to the spindle midzone (i.e. the overlapping plus ends of the central spindle microtubules) [4], [12], [13].

Conversely, Polo and Aurora B kinases are necessary for successful cytokinesis and, consistent with this, they both translocate to the spindle midzone after anaphase onset [14], [15]. The exact role of these kinases as well as their targets during cytokinesis, however, are not fully understood, because their requirement early during cell division complicates the analysis of their functions after anaphase onset. MKLP1 is known to be a target of both Polo-like kinase 1 (Plk1) and Aurora B kinase, although some controversy exists about the exact phosphorylation sites [16], [17], [18]. In *Drosophila*, Pav has been described to interact with Polo kinase and they appear mutually dependent for localization on the central spindle [6], [19]. Little is known, however, about Pav phosphorylation by either Polo or Aurora-B.

Here we investigate the dynamics of Polo kinase during cytokinesis in *Drosophila*. We show that GFP-tagged Polo undergoes a bi-phasic accumulation at the central spindle after anaphase onset, first localizing to the spindle midzone and then spreading over the entire length of the central spindle microtubules. We also demonstrate that Polo recruitment to the spindle midzone does not require Pav, but a complex formed by Fascetto (Feo) and Klp3A, the *Drosophila* homologue of KIF-4 [13], [20], [21]. Finally, we show that Polo co-localizes with Feo and Klp3A and that these two MAPs form a complex in vivo.

## Results

### Polo::GFP exhibits dynamic behavior during cytokinesis

**Figure 1:**
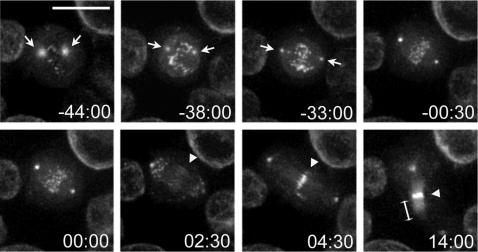
Polo::GFP dynamics during cell division. Selected images from time-lapse recordings of *Drosophila* S2 cells expressing a Polo::GFP transgene. The arrows indicate the centrosomes and the arrowheads the spindle midzone. The bracket marks the diffused localization of Polo::GFP along the central spindle microtubules. Time is in min:sec relative to anaphase onset. Bar, 10 µm.

To investigate Polo dynamics during cell division we generated a cell line stably expressing a C-terminal GFP tagged version of this kinase. Polo::GFP initially accumulated on centrosomes and astral microtubules during prophase (Figure 1, Movie S1), but as soon as the nuclear envelope started to break down, it could also be observed on kinetochores (Figure 1; -44:00 time point). Polo::GFP then accumulated inside the nucleus reaching a maximum at approximately 36 minutes before anaphase onset (±4 minutes; n = 14), indicating that the nuclear envelope was completely fenestrated (Figure 1; -38:00 time point). About 5 minutes later the two centrosomes had migrated to opposite poles (Figure 1; -33:00 time point). Proper chromosome congression and alignment was achieved about half an hour later when anaphase started (Figure 1; -00:30 and 00:00 time points). Polo::GFP began to localize to the spindle midzone 2 and a half minutes after anaphase onset (±301 seconds; n = 14) (Figure 1; 02:30 time point) and continued to accumulate during cleavage furrow formation and ingression (Figure 1; 04:30 and 14:00 time points and Movies S1 and S2). Interestingly, when the midbody formed, Polo::GFP also spread along the entire length of the central spindle microtubules (Figure 1; 14:00 time point). As the behavior of Polo::GFP in these cells appeared indistinguishable from that of a similar transgene in embryos or from antibody stainings of fixed preparations [22], [23], we believe that this distribution accurately conveys the dynamics of Polo kinase localization during cell division.

### Polo::GFP fails to localize to the spindle midzone after *feo* or *klp3A* RNAi

**Figure 2:**
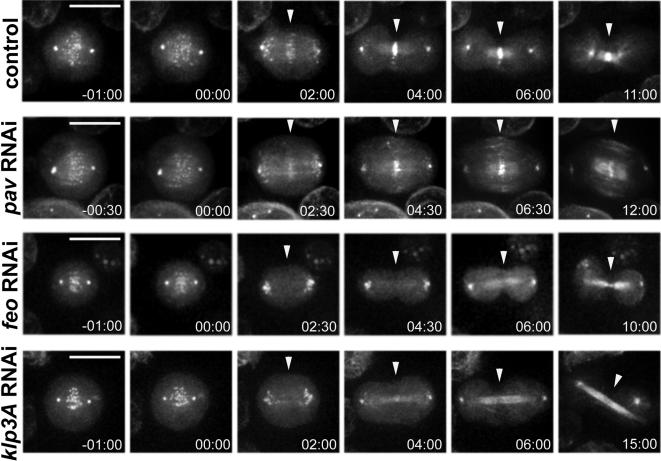
Polo::GFP does not localize to the spindle midzone after depletion of Feo or Klp3A. Selected images from time-lapse recordings of *Drosophila* S2 cells expressing a Polo::GFP transgene after RNAi depletion of Pav (*pav* RNAi), Feo (*feo* RNAi) and Klp3A (*klp3A* RNAi). Cells were treated for 48 hours with dsRNAs directed against Pav or 72 hours with dsRNAs directed against Feo and Klp3A or with no dsRNA (control). The arrowheads indicate the spindle midzone. Time is in min:sec relative to anaphase onset. Bar, 10 µm.

We used the cell line described above to analyze Polo dynamics after depleting the kinesin Pav by RNAi. As previously described for other cell lines, Pav RNAi knockdown caused cytokinesis failure after 48 hours of treatment and no cleavage furrow formation or ingression was observed (Figure 2; Movie S3) [24]. Unexpectedly, Polo::GFP localized normally at the spindle midzone from 2 and a half minutes after anaphase onset and continued to accumulate even in the absence of furrow formation and ingression in all *pav* RNAi cells examined (n = 10) (Figure 2; *pav* RNAi; from the 02:30 time point onwards). This is in contrast with previous observations that Pav is required for proper localization of Polo to the central spindle [6]. These reports, however, were based only on observations made from fixed preparations, which may have not provided an accurate examination of Polo dynamics during cytokinesis.

These results prompted us to analyze Polo::GFP localization to the central spindle after RNAi knock-down of several kinesins and MAPs known to be required for cytokinesis. In this way, we found that Polo::GFP consistently failed to localize to the spindle midzone in cells (n = 12) treated for 72 hours with dsRNA against the MAP Feo (Figure 2 and Movie S4). Two distinct dsRNAs were used in these experiments to minimize off target effects. Polo::GFP localized normally to centrosomes and kinetochores during pro-, meta- and ana-phase (Figure 2; *feo* RNAi; time points –01:00, 00:00 and 02:30), but no fluorescence was observed at the spindle midzone after anaphase onset (Figure 2; *feo* RNAi; time point 02:30), or after furrow formation and ingression (Figure 2; *feo* RNAi; from the 04:30 time point onwards). Interestingly, Polo::GFP was still able to spread along the central spindle microtubules in late telophase *feo* RNAi cells (Figure 2; *feo* RNAi; 06:00 and 10:00 time points) and, consistent with this finding, we have recently identified another MAP necessary for this localization (VA, PPD, KSL and DMG; manuscript in preparation). As previously reported for other cell lines, RNAi depletion of Feo in Polo:GFP cells caused late cytokinesis failure and the central spindle appeared much less robust than in control cells [13]. The increase in the number of multinucleate cells, however, appeared less severe in our experiments (7–8 fold; Fig. S1B) than previously published [13]. Western blot analysis confirmed an almost complete depletion of Feo protein and the ratio of multinucleate cells was independent of the presence of either the Polo::GFP or Pav::GFP transgene (Fig. S1). Thus, we conclude that this disparity may be either due to a difference in the cell type or to off-target RNAi effects.

In mammals, PRC1 depends on its interaction with KIF-4 in order to translocate to the plus ends of the central spindle microtubules [4]. We therefore depleted the *Drosophila* homologue of KIF-4, Klp3A, in Polo::GFP cells [20]. We were unable to obtain a complete depletion of Klp3A in Polo::GFP cells even after 96 hours of treatment (Fig. S1A). Nonetheless, after this treatment the cells appeared blocked in prometaphase (data not shown), a phenotype already described for *klp3A* RNAi cells [25], indicating that the functions of this kinesin were impaired under our conditions. By filming *klp3A* RNAi cells 72 hours after RNAi treatment, we were able to avoid this prometaphase block and found that, as for *feo* RNAi cells, little or no accumulation of Polo::GFP on the spindle midzone could be observed after anaphase onset in all examined cells (n = 7) (Figure 2; *klp3A* RNAi; from the 02:00 time point onwards and Movie S5). It is noteworthy that the central spindle in these cells appeared long and thin as already described in the case of Feo inactivation [13].

In conclusion, our results indicate that Polo kinase requires Feo and Klp3A to localize to the spindle midzone and not the motor protein Pav.

### Pav accumulates to the spindle midzone in *feo* RNAi cells

**Figure 3:**
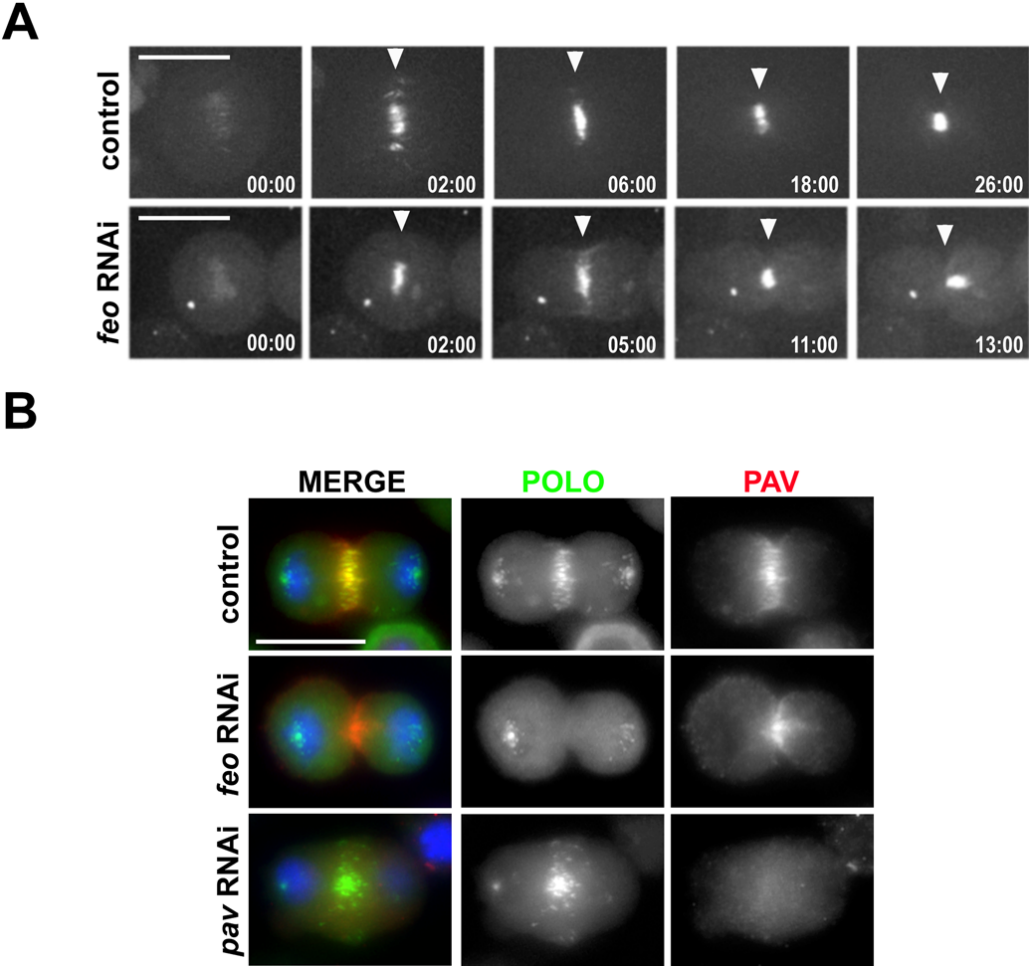
*feo* RNAi specifically disrupts Polo localization to the spindle midzone. (A) Pav::GFP accumulates to the spindle midzone after *feo* RNAi. Selected frames from a time-lapse series showing PAV::GFP localization in S2 cells after depletion of Feo. Cells were treated for 72 hours with dsRNAs directed against Feo and then recorded to visualize Pav dynamics during cytokinesis. The white arrowheads mark the spindle midzone. Time is in min:sec relative to anaphase onset. Bar, 10 µm. (B) Simultaneous detection of Polo::GFP and Pav after RNAi depletion of Pav (*pav* RNAi) and Feo (*feo* RNAi). Cells expressing Polo::GFP were treated for 48 hours with dsRNAs directed against Pav or 72 hours with dsRNAs directed against Feo or with no dsRNA (control). The cells were then fixed and stained to detect GFP (green in the merged panels), DNA (blue in the merged panels) and Pav (red in the merged panels). Bar, 10 µm.

The effects on Polo::GFP behavior observed after depletion of Feo could be caused by the general defect in the assembly and/or organization of the central spindle observed in these cells (Fig. 2) [13]. This seems unlikely, however, because a previous study indicated that other proteins known to accumulate on the spindle midzone localized correctly in *feo* RNAi cells [13]. These authors reported that, in fixed preparations of *feo* RNAi cells, the localization of Pav and the MAP Asp (Abnormal spindle) appeared normal in late anaphase but were then diffuse in late telophase. To confirm and extend these findings, we filmed cells expressing Pav::GFP during cytokinesis after depletion of Feo. Pav::GFP accumulated at the spindle midzone two minutes after anaphase onset in both control and *feo* RNAi cells (n = 7 and n = 6, respectively) and compacted as the furrow ingressed (Figure 3A; from 02:00 time point onwards; Movies S6 and S7). After complete ingression of the cleavage furrow, however, Pav::GFP localization appeared less compacted, probably as a result of a disorganized central spindle (Figure 3A; *feo* RNAi; 13:00 time point).

This result was also confirmed by immunostaining experiments where Pav and Polo::GFP were simultaneously detected after depletion of either Feo or Pav. As shown in Fig. 3B, Pav accumulated normally to the spindle midzone in *feo* RNAi cells whereas the Polo::GFP signal was totally absent (Fig. 3B, middle panels). Conversely, Polo::GFP localized to the spindle midzone in cells lacking any detectable Pav (Fig. 3B, bottom panels). Altogether, these findings indicate that the absence of Polo::GFP on the spindle midzone of *feo* RNAi cells is unlikely to be a secondary effect due to a poorly formed central spindle.

### Polo co-localizes with the Feo/Klp3A complex during cytokinesis

**Figure 4:**
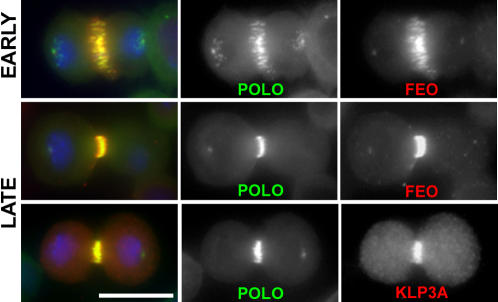
Polo co-localizes with Feo and Klp3A during cytokinesis. Localization of Feo and Klp3A during early and late telophase in S2 cells expressing Polo::GFP. Cells were fixed and stained to detect GFP (green in the merged panels), DNA (blue in the merged panels) and either Feo or Klp3A (red in the top and bottom merged panels respectively). Bar, 10 µm.

To determine if Polo co-localized with Feo and Klp3A, we stained cells expressing Polo:GFP with Feo and Klp3A antibodies. As shown in Figure 4, Polo::GFP shows a pronounced overlap with both Feo and Klp3A during late anaphase/early telophase. We then investigated if the three proteins formed a complex in vivo. To allow affinity purification, we generated stable cell lines expressing Feo tagged at its C-terminal end with two IgG binding domains of Protein A (PtA). We simultaneously generated a Feo::GFP cell line to ensure that C-terminal tagging did not alter Feo localization. This GFP tagged variant showed proper localization to the spindle midzone during cytokinesis but a weak signal was also detected around the mitotic spindle in prometaphase and metaphase (Figure S2[Supplementary-material pone.0000572.s002]). This distribution was not described before [13], but it is similar to PRC1 localization in mammalian cells [26].

**Figure 5:**
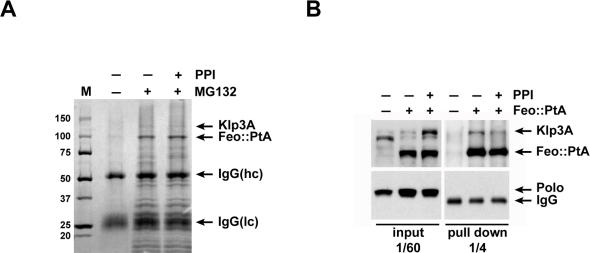
Feo and Klp3A form a complex in S2 cells. (A) Colloidal coomassie stained gel of Feo::PtA purifications from cells treated with (+) or without (−) the proteasome inhibitor MG132 and in the presence (+) or absence (−) of PPI in the lysis buffer. Feo::PtA, Klp3A and the IgG heavy (hc) and light (lc) chains are indicated. The numbers on the left indicate the size in kD of the molecular weight marker. (B) Western blots of the pull down assays from cells expressing Feo::PtA (+) or only PtA (−). The Feo::PtA purifications were performed in the presence (+) or absence (−) of PPI in the lysis buffer. The same membrane was first incubated with a mouse monoclonal Polo antibody (bottom; which also detected the IgG heavy chain) and subsequently with a Klp3A rabbit antibody (top) that cross-reacted with the IgG binding domains present in Feo::PtA.

Although we sought to co-purify Feo::PtA in complex with its mitotic partners, no protocols are currently available for synchronizing *Drosophila* tissue culture cells at different mitotic stages. We have, however, discovered that treatment with the proteasome inhibitor MG132 increases the mitotic index of S2 cells from 3% to approximately 10%. We therefore treated Feo::PtA cells with MG132 and then affinity-purified the tagged protein and interacting partners as previously described [27]. Since phosphorylation/dephosphorylation is known to be important for Feo binding activity we performed two parallel purifications, with and without phosphatase inhibitors (PPI) (Figure 5A). Treatment with MG132 led to the purification of several interacting proteins and the purification patterns with or without PPI were slightly different, suggesting that the phosphorylation status of Feo may indeed be important for its interaction with other proteins (Fig. 5A). The proteins present in these purifications were identified by tandem mass spectrometry and, as expected, Klp3A was one of the major components (Fig. 5A). This result was also confirmed by Western blot analysis (Fig. 5B). Interestingly, less Klp3A was pulled down in the presence of PPI (Fig. 5B), suggesting that the interaction between these two MAPs could require dephosphorylation.

Unexpectedly, Polo kinase was neither detected by Western blot nor identified by mass spectrometry amongst the proteins interacting with Feo::PtA (Fig. 5A, B). We were still unable to identify Polo interacting with Feo::PtA when alternative buffer conditions were used and Polo was over-expressed (data not shown). Similarly, no interaction was detected using Polo as a bait and immuno-precipitation experiments using either Polo or Feo antibodies with both cell and embryo extracts also proved unsuccessful (data not shown). These results were very surprising considering our previous findings (Fig. 2 and 4), but could be explained if Polo does not bind directly to the Feo/Klp3A complex. Alternatively, our failure to detect the interaction might also result from our inability to synchronize *Drosophila* tissue culture cells at cytokinesis (see Discussion below).

## Discussion

Since the discovery of its first member in *Drosophila* [28], the conserved family of Polo-like kinases has been the object of extensive studies in several eukaryotic systems [15], [29]. Not much attention, however, has been devoted to the study of the function of these kinases during cytokinesis, mainly because their inactivation causes an arrest at early stages during cell division. In this work, we analyzed the dynamics of Polo kinase during cytokinesis in *Drosophila* tissue culture cells and demonstrated that the Feo/Klp3A complex , but not Pav, is necessary for its recruitment to the spindle midzone (Fig. 2). This is in contrast with previous reports that indicated that in mammals Plk1 phosphorylates and interacts with MKLP1and that in flies Polo localization to the central spindle depends on the motor protein Pav [6], [18], [30]. The studies in *Drosophila*, however, did not employ time-lapse microscopy and were probably misled by the absence of cleavage furrow ingression and early collapse of the central spindle observed after Pav RNAi (Fig. 2). Furthermore, a recent report demonstrated that Plk-1 localization to the central spindle requires the mammalian orthologue of Feo, PRC1 [1]. This is in perfect agreement with the results reported here and indicates that this mechanism has been conserved during evolution.

Our experiments reveal that Feo and Klp3A form a complex during cytokinesis and their interaction appears to be regulated by dephosphorylation (Fig 5), similar to the reported interaction between PRC1 and KIF4 in mammals [4]. Polo co-localizes with Feo and Klp3A in vivo, but surprisingly was not identified in our numerous affinity purification and immuno-precipitation experiments (Fig. 5 and data not shown). Two possible explanations exist: (1) Polo does not directly interact with the Feo/Klp3A complex, or (2) the interaction does occur, but it is very weak and/or transient and therefore difficult to detect in unsynchronized *Drosophila* tissue culture cells. The strong evidence that in HeLa cells Plk1 directly binds PRC1 favors the latter hypothesis [1]. Neef et al. reported that Plk1 phosphorylates PRC1 in order to create its own docking sites [1]. The PRC1 residues targeted by Plk1, however, are not conserved in the *Drosophila* counterpart Feo (data not shown), suggesting that the mechanism in flies could be slightly different. Consistent with this, human Plk1 does not localize to the central spindle when heterologously expressed in *Drosophil*a cells and embryos [23]. Further studies are needed to determine whether Polo phosphorylates Feo and to identify potential target sites.

Cleavage furrow formation and ingression are unaffected in *feo* RNAi cells (Figs 2 and 3) [13], implying that Polo localization to the spindle midzone is not essential for furrowing. Polo also shows a diffuse distribution on the central spindle in late telophase that is independent of Feo (Figs. 1 and 2), but which requires another MAP recently identified in our laboratory. Depletion of both MAPs prevents Polo localization along the entire length of the central spindle, but does not cause a further increase in cytokinesis failure relative to Feo depletion alone (VA, PPD, KSL and DMG, manuscript in preparation). Together these data indicate that furrowing occurs normally in the absence of Polo on the central spindle. Furrow ingression is driven by the assembly and contraction of actomyosin filaments at the equatorial cortex and several observations indicate that this cytoskeletal rearrangement is orchestrated by the small GTPase RhoA [3], [31]. Recent studies in budding yeast and vertebrate cells have identified a role for Polo kinases in regulating Rho GTPase activity during cytokinesis [32], [33], [34]. It is not known if the same occurs in *Drosophila*, but our results do not support such a role for Polo kinase. We cannot exclude, however, the possibility that a cytoplasmic pool of Polo could still be recruited to the equatorial cortex even in the absence of the two MAPs necessary for its localization to the central spindle. Consistent with this hypothesis, Polo is required for proper localization of the myosin regulatory light chain at the cortex before anaphase onset [23].

In conclusion, it is evident that the basic molecular feature of Polo recruitment to the spindle midzone during cytokinesis has been conserved during evolution as both the fly and human kinases require a complex composed by two conserved MAPs. Quite intriguingly, however, the mechanisms in the two organisms also appear to have acquired slightly diverged characteristics as human Plk1 and PRC1 have evolved an interaction mechanism that it is clearly different from *Drosophila*. It would be interesting to determine when and how these two systems diverged and perhaps studies in the nematode *C. elegans* could provide some answers. Certainly, the identification of Polo targets during cytokinesis in both flies and mammals will be crucial if we are to understand whether the downstream events regulated by this important family of mitotic kinases have also been conserved.

## Materials and Methods

### Cell culture, RNAi treatments, DNA constructs and Generation of Stable Cell Lines

The DMEL strain of S2 cells (Invitrogen) was used in all the experiments. These cells have been adapted to grow in Serum Free Medium (SFM). Generation of dsRNAs and RNAi treatments were performed as described [24], [35]; two distinct dsRNAs were used for Feo knock-downs with identical results (data not shown). Oligonucleotide sequences are available upon request.

Plasmids expressing Polo::GFP, Feo::GFP and Feo::PtA under the control of the Actin5C promoter were constructed used Gateway® technology (Invitrogen) as described [24]. Stable cell lines were generated using blasticidin selection as previously published [24].

### Microscopy

Immunocytochemistry was carried out as previously described [35]. The Feo, Klp3A and Pav antibodies and their respective working dilution were as described [13], [20], [36]. For time-lapse experiments, S2 cells were plated on No. 1 1/2 thickness acid cleaned coverslips 72 hours after RNAi treatment. Imaging was performed on a Zeiss Axiovert 200 microscope with a 100x (N.A. 1.4) lens. Specimens were maintained at 25°C via a dual stage and objective heating unit (Tempcontrol 37-2 digital; Zeiss). At 30-second intervals a z-series of 10, 1-µm sections was captured using a Perkin Elmer Ultraview RSIII spinning disk confocal head. All images shown are maximum intensity projections.

### Protein Affinity Purifications, Western blot and Mass Spectrometry

Protein-A affinity purification was performed as previously described with minor modifications [27]. The proteasome inhibitor MG132 (Sigma) was added to the SFM medium at a final concentration of 20 µM and cells incubated for 16 hours before collection. After collection, 6x10^8^ cells were resuspended in lysis buffer with or without a cocktail of phosphatase inhibitors (Sigma). The proteins were then separated on an 8–16% acrylamide gel and the bands excised and analyzed by LC-MS/MS. Reverse phase liquid chromatographic separation of peptides was carried out using a PepMap C18 reverse phase, 75 mm i.d., 15-cm column (LC Packings, Amsterdam) on a Eksigent nano LC system attached to a LTQ-Orbitrap (Thermo Finnigan) mass spectrometer. The MS/MS fragmentation data achieved was used to search the National Center for Biotechnology Information and Flybase databases using the MASCOT search engine (http://www.matrixscience.com). Probability-based MASCOT scores were used to evaluate identifications. Only matches with P < 0.05 for random occurrence were considered significant.

For pull-down assays, 5x10^7^ cells were collected and processed as above and the proteins fractioned on an 8–16% SDS-PAGE gel and transferred onto a PVDF membrane as previously described [35]. Feo and Klp3a antibodies were used at a dilution of 1:5000 and Polo antibody at a dilution of 1:200.

## Supporting Information

Figure S1 Depletion of Feo causes similar cytokinesis defects in cells expressing different transgenes.(A) Western blot analysis of protein levels after RNAi treatment. Polo::GFP cells were incubated with dsRNAs directed against Feo (feo RNAi), Klp3A (klp3A RNAi) or no dsRNA as a control. After 72 or 96 hours the proteins were extracted and separated on a 10% SDS gel, transferred onto a PVDF membrane and probed with antibodies against Feo, Klp3A and α-tubulin. (B) Increase of multinucleate cells after feo RNAi in cells expressing Polo::GFP, Pav::GFP or no transgene (control). Results are means and s.d. from 3 distinct experiments using two different dsRNAs. More than 1000 cells were counted in each experiment.
(6.05 MB TIF)

Figure S2 Localization of Feo::GFP during mitosis. Cells were fixed and stained to detect Feo::GFP (green in the merged panels), DNA (blue in the merged panels) and Tubulin (red in the merged panels). Bar, 10 µm
(3.13 MB TIF)

Movie S1 Polo::GFP behavior during cell division. This movie shows Polo::GFP dynamics during mitosis, from prophase to cytokinesis. See Figure 1 for details. All sequences were captured at 30 second intervals. Playback rate is 6 frames per second (FPS).
(11.40 MB AVI)

Movie S2 Polo::GFP dynamics during cytokinesis in control cells. This movie shows Polo::GFP dynamics from prometaphase in a control cells. See Figure 2 for details. All sequences were captured at 30 second intervals. Playback rate is 6 frames per second (FPS).
(9.90 MB AVI)

Movie S3 Pav is not necessary for Polo recruitment to the spindle midzone. Polo::GFP cells were treated with dsRNA directed against Pav for 48 hours and then filmed from prometaphase. See Figure 2 for details. All sequences were captured at 30 second intervals. Playback rate is 6 frames per second (FPS).
(5.99 MB AVI)

Movie S4 Polo is not recruited to the spindle midzone after feo RNAi. Polo::GFP cells were treated with dsRNA directed against Feo for 72 hours and then filmed from prometaphase. See Figure 2 for details. All sequences were captured at 30 second intervals. Playback rate is 6 frames per second (FPS).
(6.10 MB AVI)

Movie S5 Polo is not recruited to the spindle midzone after klp3A RNAi. Polo::GFP cells were treated with dsRNA directed against Klp3A for 72 hours and then filmed from prometaphase. See Figure 2 for details. All sequences were captured at 30 second intervals. Playback rate is 6 frames per second (FPS).
(5.50 MB AVI)

Movie S6 Pav::GFP behavior during cytokinesis. This movie shows Pav::GFP dynamics from prometaphase in a control cells. See Figure 3 for details. All sequences were captured at 30 second intervals. Playback rate is 6 frames per second (FPS).
(5.91 MB AVI)

Movie S7 Pav is recruited to the spindle midzone after feo RNAi. Pav::GFP cells were treated with dsRNA directed against Feo for 72 hours and then filmed from prometaphase. See Figure 3 for details. All sequences were captured at 30 second intervals. Playback rate is 6 frames per second (FPS).
(2.94 MB AVI)
