# Supplementary figures and images for: Recruitment of Polo Kinase to the Spindle Midzone during Cytokinesis Requires the Feo/Klp3A Complex
Source: PLoS One. 2007 Jun 27;2(6):e572. doi: 10.1371/journal.pone.0000572 (PMC1894651; doi:10.1371/journal.pone.0000572)

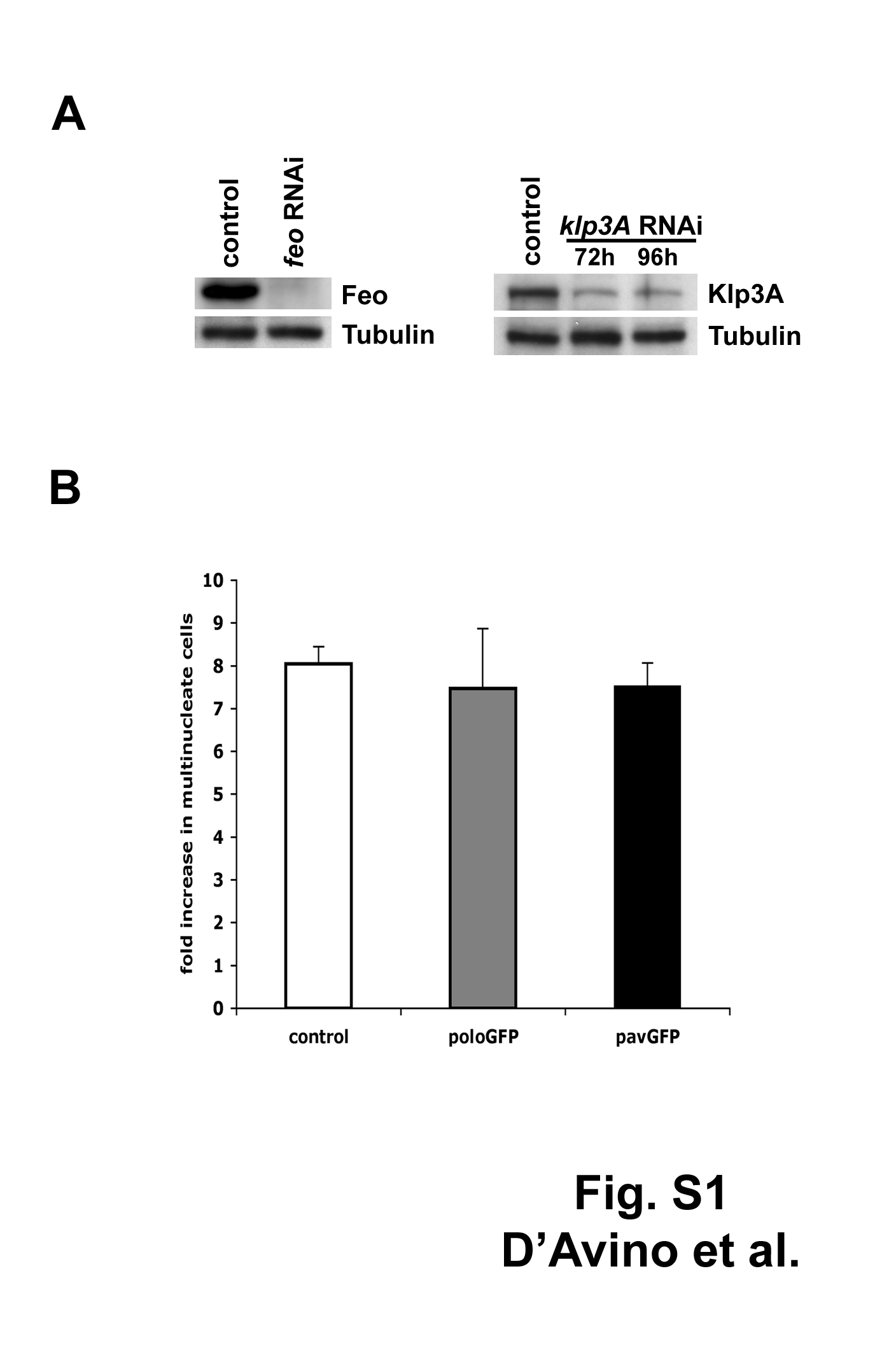

Supplement: Figure S1 — Depletion of Feo causes similar cytokinesis defects in cells expressing different transgenes.(A) Western blot analysis of protein levels after RNAi treatment. Polo::GFP cells were incubated with dsRNAs directed against Feo (feo RNAi), Klp3A (klp3A RNAi) or no dsRNA as a control. After 72 or 96 hours the proteins were extracted and separated on a 10% SDS gel, transferred onto a PVDF membrane and probed with antibodies against Feo, Klp3A and α-tubulin. (B) Increase of multinucleate cells after feo RNAi in cells expressing Polo::GFP, Pav::GFP or no transgene (control). Results are means and s.d. from 3 distinct experiments using two different dsRNAs. More than 1000 cells were counted in each experiment. (6.05 MB TIF) [file pone.0000572.s001.tif]

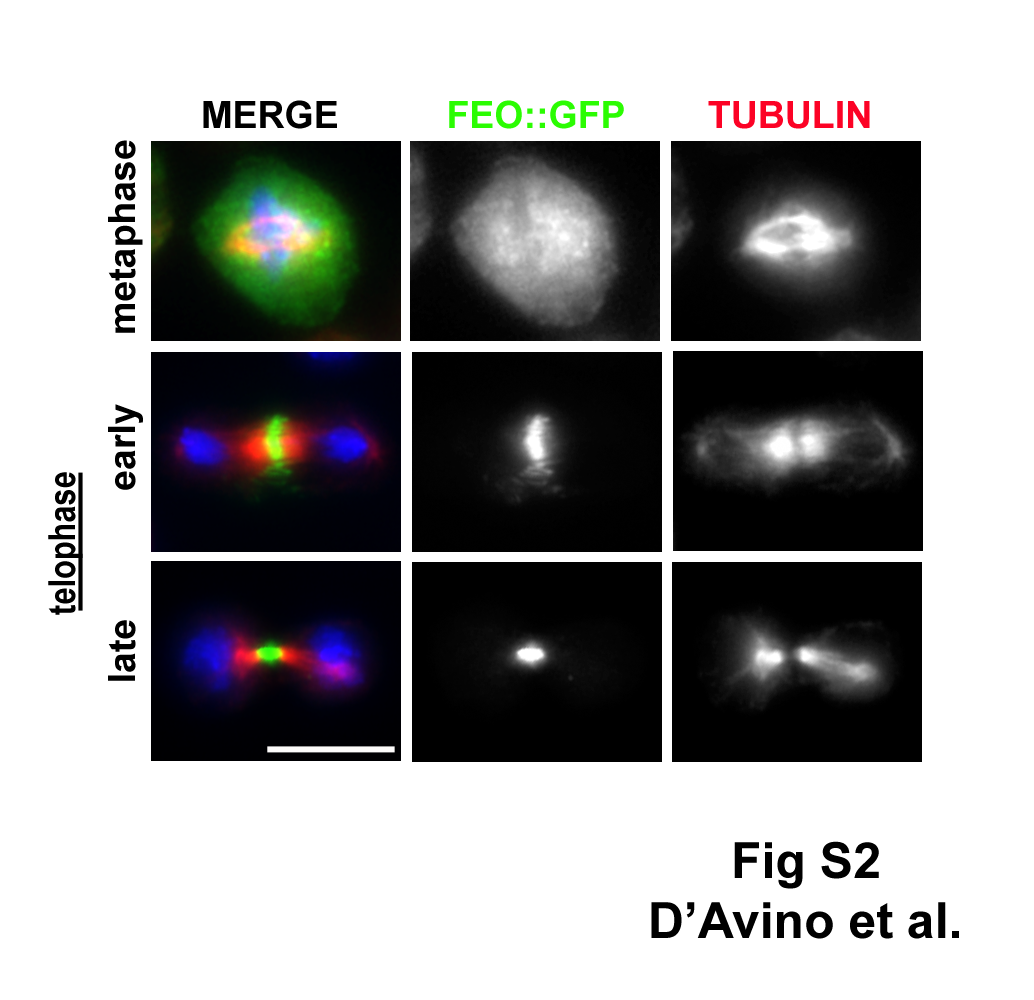

Supplement: Figure S2 — Localization of Feo::GFP during mitosis. Cells were fixed and stained to detect Feo::GFP (green in the merged panels), DNA (blue in the merged panels) and Tubulin (red in the merged panels). Bar, 10 µm (3.13 MB TIF) [file pone.0000572.s002.tif]
